# Hypertrophic Olivary Degeneration and Movement Disorder in a Patient with Familial Creutzfeldt-Jakob Disease

**DOI:** 10.7759/cureus.10854

**Published:** 2020-10-08

**Authors:** Andre Granger, Shashank Agarwal, Andres Andino, Patrick Kwon, Elina Zakin

**Affiliations:** 1 Department of Neurology, New York University Grossman School of Medicine, New York, USA

**Keywords:** creutzfeldt jakob disease, familial, movement disorder, hypertrophic olivary degeneration, rapidly progressive dementia

## Abstract

A 38-year-old male presented with a three-week history of bilateral lower extremity choreiform movements. History included sleep abnormalities, rushed and unintelligible speech, with delusions two to six months prior to presentation. He also developed mild dysphagia, staring spells, and anterograde amnesia. On examination, he had pressured speech, asynchronous cycling movements of the bilateral lower extremities persisting during sleep, occasional ballistic movements of the upper extremities, and ataxia. Magnetic resonance imaging (MRI) of the brain showed high cortical signal change in bilateral parieto-occipital cortices with evidence of medullary olive hypertrophy bilaterally. Electroencephalography showed generalized slowing without periodic spikes. Cerebrospinal fluid was positive for protein 14-3-3 and real-time quaking-induced conversion. Genetic testing was positive for autosomal dominant prion protein gene (PRNP) genetic mutation. The patient passed away three months after discharge.

This case provides previously undescribed imaging and movement abnormalities in a patient with familial Creutzfeldt-Jakob disease (CJD), and suggests that CJD should not be removed from the differential in patients with these atypical findings.

## Introduction

Human transmissible spongiform encephalopathies are a group of disorders that cause rapidly progressive dementia, and ultimately death, due to aggregation of misfolded prion proteins. This class of neurodegenerative conditions is usually sporadic but may also be inherited or acquired [1]. Patients with Creutzfeldt-Jakob disease (CJD) can present with a prodrome that includes symptoms of headaches, anxiety, behavioral changes, memory loss, speech problems, and insomnia. These symptoms progress to include rapid cognitive deterioration, extrapyramidal symptoms, and later death [2]. Movement abnormalities previously described in CJD include spasticity, rigidity, myoclonic jerks, and ataxia [2]. Other less common movement disturbances include alien-limb phenomenon, athetosis, and chorea [3,4].

A number of variants of CJD have been defined based upon focal neurologic findings reflecting predominant involvement of individual brain regions. Examples of these include forms with mainly visual (Heidenhain variant) [5], cerebellar (Oppenheimer-Brownell variant) [6], and thalamic features [7].

## Case presentation

A 38-year-old man with no past medical history presented with a three-week history of abnormal lower extremity movements. Six months prior to presentation, he developed insomnia and would sleep for 3-4 hours each night. He sought medical attention and was prescribed trazodone, which was stopped after a week due to morning headaches. Two months later, his speech became abnormal, noted to be rushed and unintelligible after the first few words. He then developed unsteadiness and was taken to the hospital where he was diagnosed with posterior reversible encephalopathy syndrome (PRES) and discharged on antihypertensives. Two months prior to presentation, he developed delusions, and worsening hypersomnia. Three weeks before his admission, he developed involuntary “stepping” movements of the bilateral lower extremities. This movement persisted during sleep and caused him significant fatigue. Other symptoms included mild dysphagia, brief staring spells, and anterograde amnesia. There was no reported history of fever, weight loss, recent travel, sick contacts, animal bites, drug use, or new medications. He worked in construction but was recently laid off due to his unsteadiness and speech abnormalities. Family history was significant for delusions and hallucinations in his mother who died at an early age due to an unknown illness.

Examination was notable for pressured speech, delayed recall of two out of three items, saccadic intrusions with square wave jerks on extraocular motion testing, alternating stepping movements of the bilateral lower extremities, and rare ballistic movements of the upper extremities (Video 1).

**Video 1:** Coarse Bilateral Lower Extremity Tremors in a Patient with Familial Creutzfeldt-Jakob Disease

Gait testing was deferred as he could no longer ambulate at the time of presentation.

**Figure 1:**
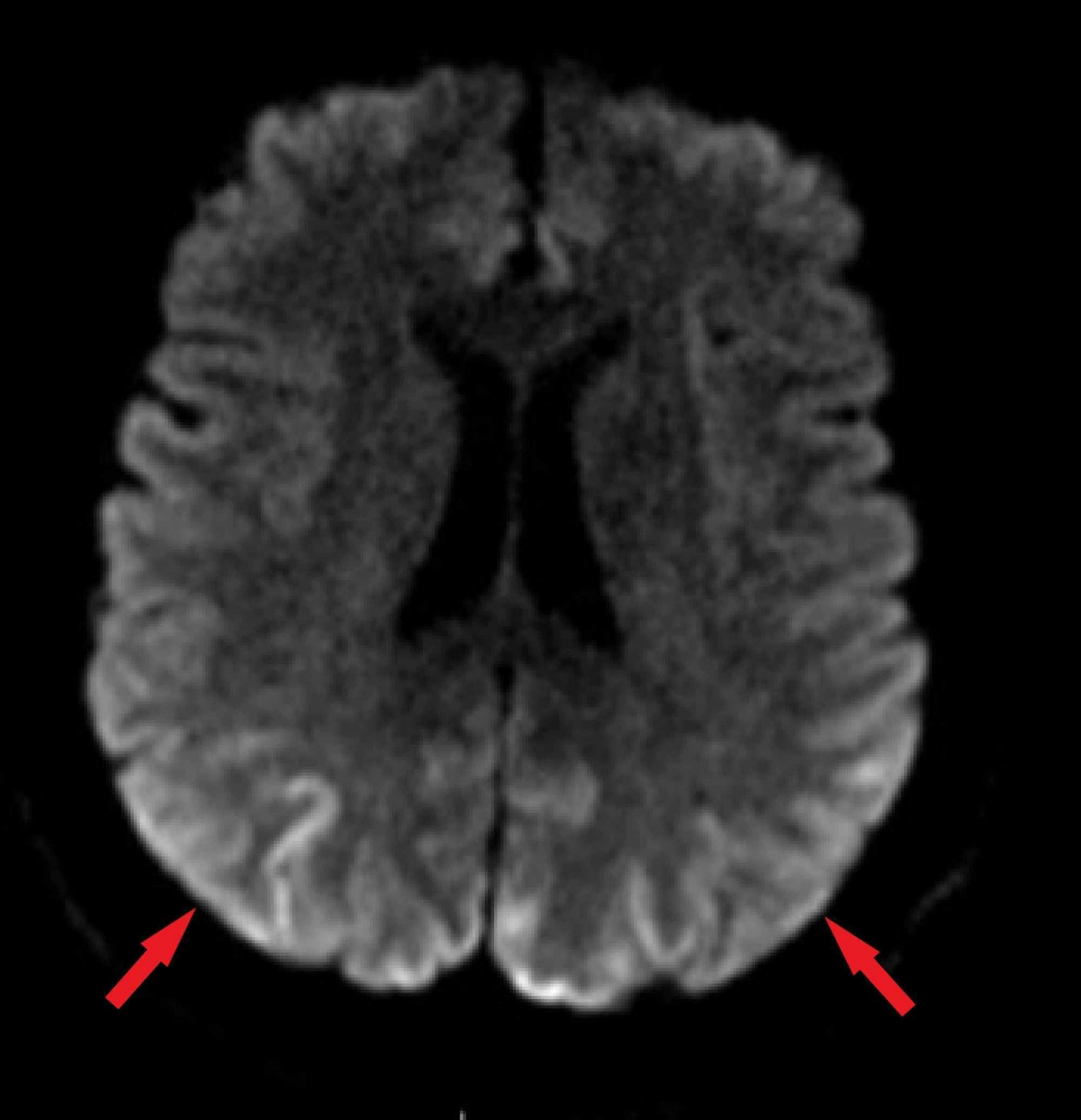
Parieto-occipital Cortical Diffusion Restriction on Axial Magnetic Resonance Imaging

**Figure 2:**
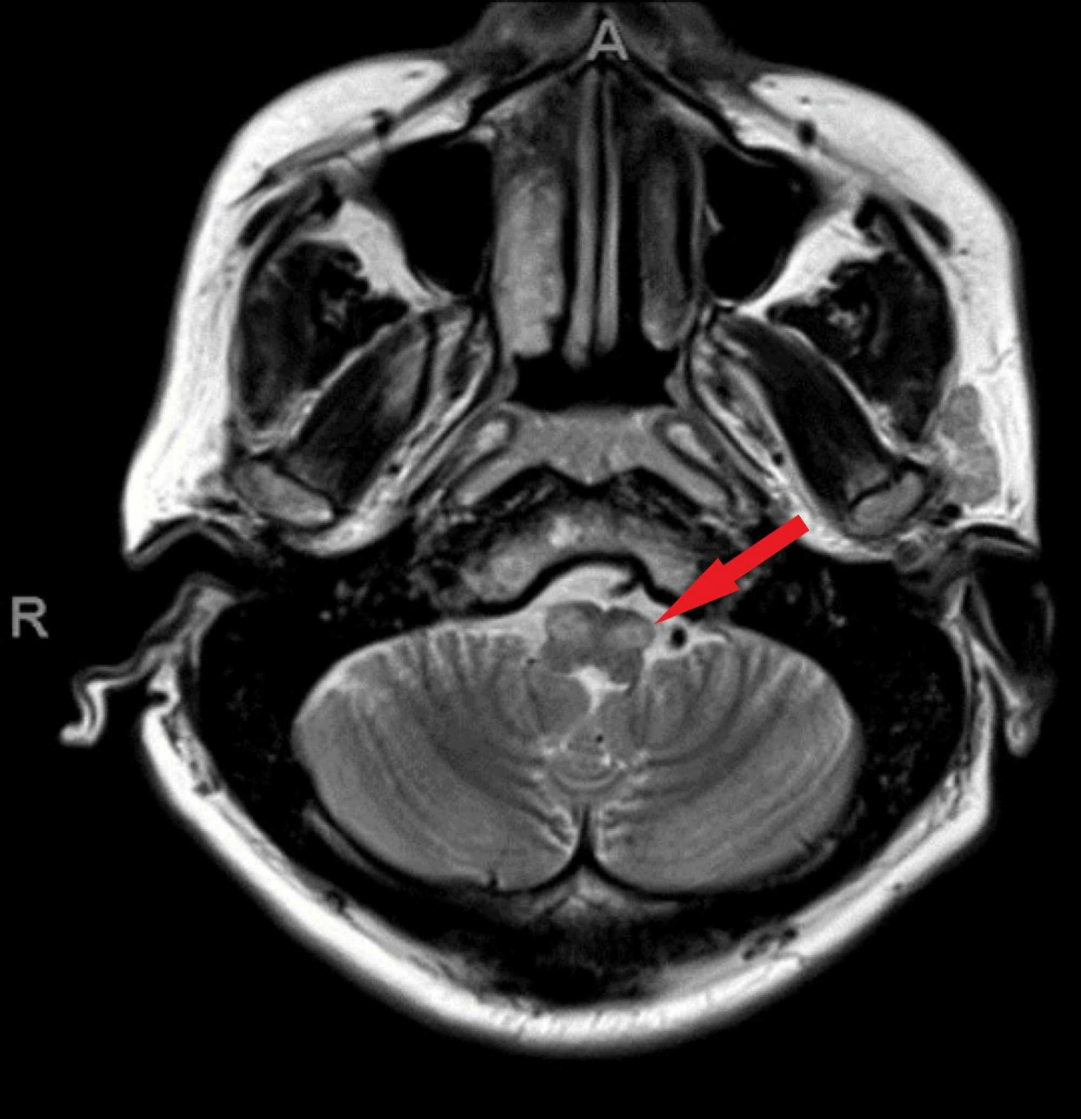
Hyperintensities Within the Inferior Olivary Nuclei on Axial T2-Weighted Magnetic Resonance Imaging

**Figure 3:**
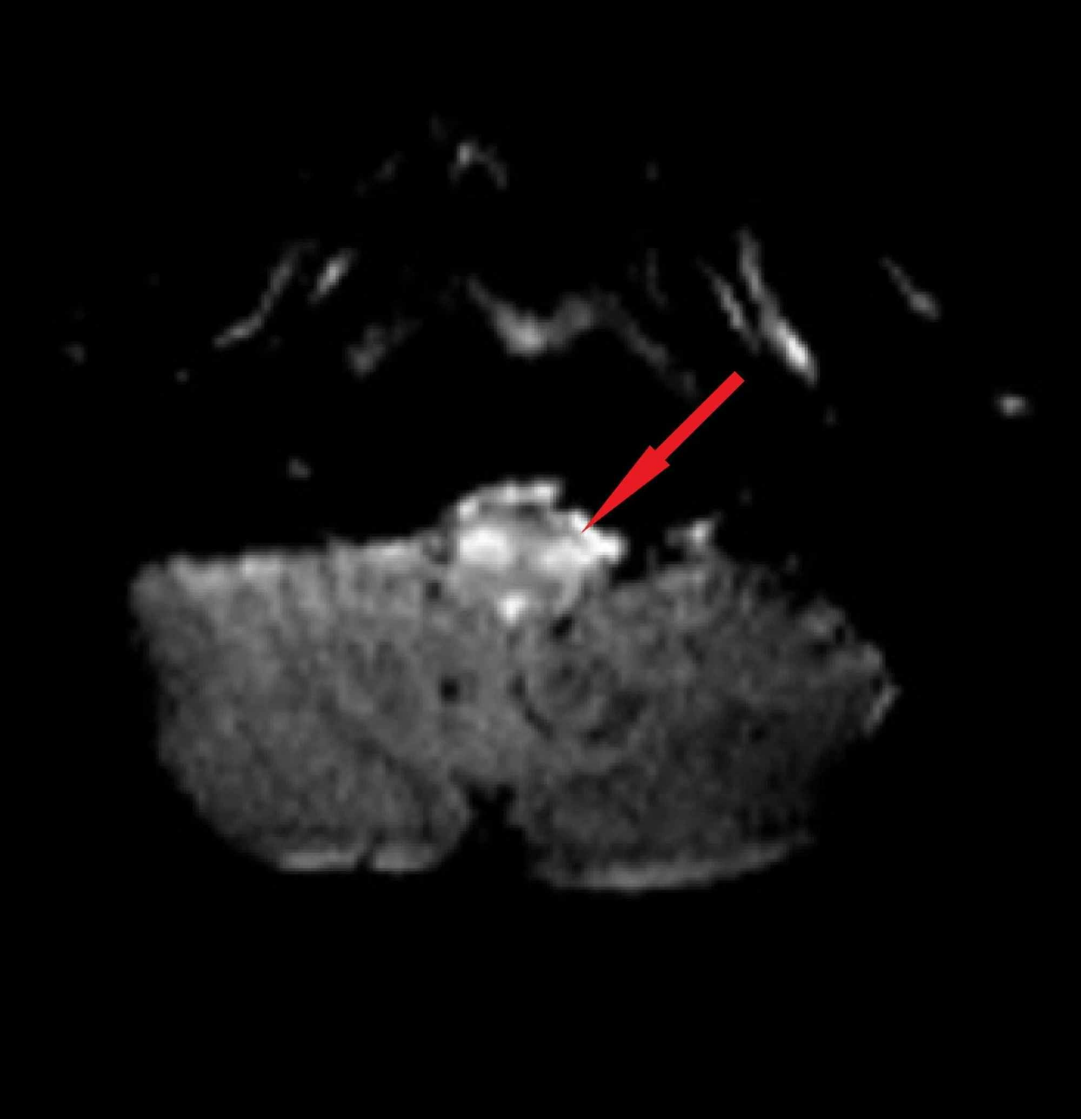
Hyperintensities in the Inferior Olivary Nuclei on Axial Fluid-Attenuated Inversion Recovery Magnetic Resonance Imaging

During his hospital stay, magnetic resonance imaging (MRI) of the brain showed high cortical signal change in bilateral parieto-occipital cortices with hypertrophic olivary degeneration (Figures 1-3).

Video electroencephalogram showed generalized slowing without periodic spikes. Organophosphate levels were mildly elevated. The infectious, inflammatory, and neoplastic workup was negative. Cerebrospinal fluid was positive for both protein 14-3-3 and real-time quaking-induced conversion. Genetic testing was positive for the autosomal dominant prion protein gene (PRNP) genetic mutation essentially confirming the diagnosis of familial CJD (fCJD). The patient’s family was counseled extensively on the poor prognosis of the disease and they opted for palliative care. The patient passed away three months after discharge. The family declined autopsy.

## Discussion

Abnormal voluntary and involuntary movements are important findings to assist in the identification of patients with possible CJD. Myoclonus, cerebellar signs, pyramidal/extrapyramidal signs, and akinetic mutism are all components of the Center for Disease Control (CDC) diagnostic checklist [8]. Myoclonus, gait disturbances, ataxia, dysmetria, and rigidity were reported in 40%-80% of patients in one retrospective study [4]. Chorea was present in 2%-11% of patients with CJD at some point during their disease course [4,9]. It appears to be a more common feature of variant CJD as 20 of 34 such patients were reported to have developed chorea later in the disease course [10].

Several MRI abnormalities have been previously reported in patients with sporadic CJD. Among them, cerebral cortical or subcortical high signal changes, and high signal intensity in the caudate nucleus and putamen are common enough to form part of the diagnostic criteria [11]. Other neuroanatomical structures that tend to be involved include the thalamus and limbic areas [12]. Findings in fCJD are less commonly discussed due to its lower incidence rates when compared to sporadic cases. In one study of six patients with fCJD, five had MRI abnormalities [13]. Vitali et al. described MRI findings that were similar to that of sporadic cases, and included abnormal intensities in the limbic, neocortical, and subcortical areas [13].

Less common variants of CJD have been described based on specific imaging findings. Visual abnormalities such as visual hallucinations, visual field deficits, cortical blindness, visual agnosia, and defects in visuospatial and color perception are characteristic of the Heidenhain variant [14,15]. These symptoms reflect optic pathway pathology and can be the initial presenting signs of this variant. Positron emission tomography (PET) scans may show hypometabolism in the occipital lobes, and pathology may show pronounced changes in the occipital lobes [5]. In the Oppenheimer-Brownell variant, patients present with ataxia that rapidly progresses, and is followed by dementia. These cases are associated with selective loss of the granule cells in the cerebellar cortex [16]. There have also been two other proposed subtypes based on predominantly cognitive or affective symptoms [6].

In the case presented earlier, there is clear involvement of the inferior olivary nuclei without basal ganglia involvement. The inferior olivary nuclei form part of the dentato-rubro-olivary pathway (Guillain-Mollaret triangle). Lesions in this pathway classically produce palatal myoclonus, in which there are rhythmic and stereotypic clonic jerks of the soft palate at a frequency of 1-3 Hz [17]. Occasionally, the eyes may be involved and pendular nystagmus may be observed. Even less commonly, the extremities can be involved. In such instances, the finding is a postural tremor that alternates in an antagonistic fashion and is in sync with the palatal tremor [17].

In this patient, there was no evidence of palatal myoclonus. However, there were repeated, stereotypical, alternating flexion-extension movements noted at bilateral hips and knees, with each limb jerking at a frequency of about 2 Hz. Similar to symptomatic palatal myoclonus, these movements did not terminate during sleep [17]. In one thalamic subtype of sporadic CJD, severe neuronal loss and gliosis in the olives, not accompanied by spongiosis, has been described [7]. Furthermore, pathological and immunohistochemical analysis has confirmed that the abnormal prion protein is prominently deposited in the inferior olivary nuclei even when there is no associated significant neuronal loss or spongiform changes [18].

Hypertrophic olivary degeneration has not been previously described in fCJD. The accompanying lower extremity movements have also never been described in fCJD. We suspect that the lesions were related to our patient’s prion disease as prion protein has been frequently found in this location, and he had no lesion in the Guillain-Mollaret triangle to explain the etiology of this finding. There was no report of olivary abnormality on his brain MRI obtained at a different facility four months earlier to suggest that this lesion was chronic. Furthermore, we postulate that his abnormal lower extremity movement disorder could be related to this imaging finding as there were characteristics that overlapped with symptomatic palatal myoclonus. A theoretical explanation of these movements utilizes the connection of the Guillain-Mollaret triangle with the rubrospinal tract. The rubrospinal tract forms part of the lateral motor system and is thought to have predominantly flexor, or anti-extensor effects [19,20]. Given that our patient had rhythmic flexor movements at the hip, an aberrantly excited rubrospinal tract could have played a role. Another possible explanation, not involving the inferior olives, would be that there was indeed dysfunction of the basal ganglia sufficient enough to lead to a movement disorder, but insufficient enough to cause MRI changes.

The limitations of this report include the fact that no pathologic specimen was available to confirm prion protein deposition in the olives. Also, the patient’s movement disorder did not include upper limb myoclonic movements during his hospital stay, which would beg the question of why the lower limbs would be selectively affected in a rubrospinal pathology. Additionally, due to the paucity of similar cases, a strong causal link cannot be made between the abnormality of the inferior olives and these movements.

## Conclusions

The diagnosis of CJD remains challenging due to the large variability in the clinical presentations. It should be one of the differential diagnoses considered in patients who present with subacute movement disorder, rapidly progressive cognitive changes, and imaging findings of hypertrophic medullary olives. More cases of fCJD need to be reported to assist in building a more comprehensive profile for this disease.
